# New Insights into the Crosstalk between NMDARs and Iron: Implications for Understanding Pathology of Neurological Diseases

**DOI:** 10.3389/fnmol.2017.00071

**Published:** 2017-03-16

**Authors:** Huamin Xu, Hong Jiang, Junxia Xie

**Affiliations:** ^1^Collaborative Innovation Center for Brain Science, Department of Physiology, Shandong Provincial Key Laboratory of Pathogenesis and Prevention of Neurological Disorders and State Key Disciplines: Physiology, Medical College of Qingdao UniversityQingdao, China; ^2^Shandong Provincial Collaborative Innovation Center for Neurodegenerative Disorders, Qingdao UniversityQingdao, China

**Keywords:** Parkinson’s disease, Alzheimer’s disease, iron, NMDA receptor, divalent metal transporter 1

## Abstract

Both iron dyshomeostasis and N-methyl-D-aspartate receptors (NMDARs)-mediated neurotoxicity have been shown to have an important role in neurological diseases such as Parkinson’s disease (PD) and Alzheimer’s disease (AD). Evidence proved that activation of NMDARs could promote iron overload and iron-induced neurotoxicity by enhancing iron importer divalent metal transporter 1 (DMT1)-mediated iron uptake and iron releasing from lysosome. Also, iron overload could regulate NMDARs-mediated synaptic transmission. This indicates that there might be a possible relationship between iron and activation of NMDARs in neurological diseases. Understanding this interaction between iron and activation of NMDARs may provide new therapeutic avenues for a more targeted neurotherapeutic strategy for these diseases. Therefore, in this review article, we will describe the dysfunction of iron metabolism and NMDARs in neurological diseases including PD and AD, and summarize the new insight into the mechanisms underlying the interaction between iron and activation of NMDARs.

## Introduction

Iron is an important cofactor in many proteins such as heme-containing proteins and iron-containing enzymes. It is required for many physiological processes for life (Zucca et al., 2015). In the central nervous system (CNS), iron also participates in myelin synthesis, development of dendritic spines in the hippocampus and synthesis of neurotransmitters including monoamine transmitters and gamma-aminobutyric acid (GABA; Li, 1998; Jorgenson et al., 2003; Todorich et al., 2009; Zucca et al., 2015; Bastian et al., 2016). However, excess iron is toxic due to its ability to produce cytotoxic hydroxyl radicals, which could cause damages to proteins, nucleic acids and cell membranes (Whitnall and Richardson, 2006). Therefore, intracellular iron metabolism is tightly regulated. There are two pathways for iron uptake: the classical transferrin (Tf)-mediated iron uptake pathway and the non-Tf bound iron (NTBI) uptake pathway (Qian and Shen, 2001). Traditionally, the Tf-transferrin receptor 1 (Tf-TfR1) pathway is considered as major pathway for cellular iron uptake in the brain. Divalent metal transporter 1 (DMT1) is identified as the main NTBI pathway, responsible for ferrous iron uptake. There are four DMT1 mRNA isoforms: N-terminus (1A, 1B) generated from alternative promoters with subsequent, exclusive splicing of the respective first exon to exon 2 and C-terminus splice variants (+iron-responsive element (IRE), −IRE) due to alternative splice mechanisms (Hubert and Hentze, 2002). DMT1 + IRE contains an IRE in the 3′-untranslated region (UTR) and DMT1 − IRE has no IRE. Additionally, 1A/DMT1 is predominantly expressed in kidney and duodenum and 1B/DMT1 is ubiquitously expressed in the peripheral organs and brain (Hubert and Hentze, 2002). Ferroportin 1 (FPN1), also known as metal transport protein1 (Abboud and Haile, 2000) or iron-regulated transporter 1 (IREG1) is the only known iron transporter responsible for cellular iron export (Ganz, 2005). Disturbance in iron metabolism plays a key role in the pathogenesis of neurodegenerative disorders such as Parkinson’s disease (PD) and Alzheimer’s disease (AD) (LaVaute et al., 2001; Lieu et al., 2001; Perez et al., 2008; Zhu et al., 2009). Elevated iron levels were found in the substantia nigra (SN) in PD patients (Sofic et al., 1988). Studies also showed that iron accumulation was observed in the hippocampus and cerebral cortex in AD patients (Piñero et al., 2000, 2001; Altamura and Muckenthaler, 2009). Increased import, decreased export or redistribution of intracellular iron might be responsible for iron metabolism disturbance in these diseases, which may increase the vulnerability of neurons to iron.

Glutamate is the major excitatory neurotransmitter in the CNS, exerting its functions by binding to different receptors. Among them, N-methyl-D-aspartate receptors (NMDARs) are cation channels that mediate entry of Na^+^ and Ca^2+^ ions and are activated by the co-agonists glutamate (or NMDA) and glycine or D-serine. There are several subunits of NMDARs: NR1, NR2A, NR2B, NR2C and NR2D and NR3. Among them, NR1 is a fundamental subunit of the receptor. Appropriate activation of NMDARs plays a critical role in physiological functions such as excitatory neurotransmission, synaptic plasticity (Carvajal et al., 2016). However, excitotoxicity induced by excessive activation of NMDARs contributes to pathological changes in the CNS (Ambrosi et al., 2014; Gonzalez et al., 2015). Influx of Ca^2+^ through NMDARs might lead to neuronal loss in several neurodegenerative diseases such as PD and AD (Hynd et al., 2004). NMDARs have both synaptic and extrasynaptic locations. One hypothesis posits that synaptic NMDARs were neuroprotective and extrasynaptic NMDARs were neurotoxic (Hardingham and Bading, 2010). The death signaling induced by extrasynaptic NMDARs or relocalization of NMDARs to extrasynaptic sites has been shown to contribute to pathology of neurodegenerative diseases (Bading, 2017). Fine-tuning might provide a promising operation to optimize the activity of the glutamatergic system in order to maintain normal function of neurons (Köles et al., 2016). Activation of NMDARs as well as iron deposits in neurological diseases including PD and AD suggest that there might be a correlation between iron deposition and activation of NMDARs. Therefore, in this article, we reviewed the studies about the involvement of iron dyshomeostasis and NMDARs-mediated neurotoxicity in PD and AD. We then described the advanced knowledge on interaction of iron and NMDARs activation. This will provide implications for understanding pathology of these neurological diseases.

## Dysfunction of Iron Metabolism and NMDARs in PD

PD is a common neurodegenerative disorder characterized by loss of dopamine (DA) neurons in the SN pars compacta (SNpc), resulting in depletion of DA in the striatum (Parkinson, 2002; Hornykiewicz, 2008). Although the etiology of PD has not been clarified until now, iron accumulation and excitatory neurotoxicity are considered as contributing factors to the etiology of PD. It has become increasingly evident that elevated iron levels in the SNpc play a key role in the degeneration of DA neurons in PD (Jiang et al., 2016; Muñoz et al., 2016). Nigral iron levels increased with age, and PD patients showed an even greater increase, which correlates with clinical PD status (Wu et al., 2014; Du G. et al., 2016). Experimental evidence also showed that iron was involved in the degeneration of SNpc dopaminergic neurons in 1-methyl-4-phenyl-1,2,3,6-tetrahydropyridine (MPTP) and 6-hydroxydopamine (6-OHDA)-induced animal models of PD (Salazar et al., 2008; Jiang et al., 2010). Mechanisms underlying iron-induced neurodegeneration of DA neurons have been reported: First, iron participates in Fenton reaction to generate ·OH, which could damage proteins, nucleic acids and cell membranes (Mohanakumar et al., 1994). Second, iron-induced α-synuclein oligomers can form ion-permeable pores in lipid bilayers and give rise to α-synuclein-dependent toxicity in neuronal cells (Kostka et al., 2008). Our previous studies also showed iron could promote α-synuclein aggregation (He et al., 2013). Third, iron overload induced mitochondrial fragmentation via increasing intracellular calcium (Ca^2+^) and activated calcineurin via Ca^2+^/calmodulin and Ca^2+^/calpain pathways. This mitochondrial fragmentation and neuronal cell death could be rescued by chelation of intracellular Ca^2+^ (Lee et al., 2016). Recently, it was reported that ferroptosis, an iron-dependent regulated cell death process, is an important cell death pathway for DA neurons in PD (Do Van et al., 2016).

A recent research showed that increase in DMT1 expression, rather than TfR1 and Fpn1 expression might be partly responsible for age-dependent increase in brain iron (Lu et al., 2016). This indicated the importance of DMT1 in age-induced iron overload. Our previous studies showed that increased expression of DMT1 + IRE and decreased expression of FPN1 were responsible for nigral-specific iron accumulation in PD models (Wang et al., 2007; Jiang et al., 2010; Song et al., 2010). DMT1 was found in the SNpc and associated predominantly with neuromelanin-containing DA neurons. It was consistently less intense in DA neurons of the ventral tegmental area (VTA) than in those of the SNpc in the same tissue sections of human brain older than 60 years (Salazar et al., 2008). In addition, postmortem studies have shown an increase of DMT1 in the SN of PD patients (Salazar et al., 2008). And a mutation in DMT1 that impaired iron transport protected rodents against parkinsonism-inducing neurotoxins MPTP and 6-OHDA (Salazar et al., 2008). Recent study demonstrated that DMT1 polymorphisms might be a risk factor for PD (Saadat et al., 2015). This indicated that higher DMT1 expression and consequently higher iron levels in nigral DA neurons might contribute to increased vulnerability of nigral DA neurons to PD-related neurotoxins (Huang E. et al., 2004). The mechanisms underlying the expression of iron transporters DMT1 + IRE and FPN1 are IRE/iron regulatory proteins (IRPs)-dependent in 6-OHDA-induced PD models and IRE/IRPs-independent in MPP^+^-treated dopaminegic cells (Zhang et al., 2009; Jiang et al., 2010). In addition, ceruloplasmin (CP) is the strongest ferroxidase to stabilize iron exporter FPN1. It was reported that the activity of CP in PD brains was reduced in SN (Ayton et al., 2013). In CP-knockout mice, iron overload was found in several tissues, including the brain (Kaneko et al., 2008). In our previous report, we also showed that decreased expression of CP in the SN was involved in the nigral iron accumulation of 6-OHDA-induced PD rats (Wang et al., 2015). These results suggested that CP might also play an important role in iron deposit in PD.

In addition, NMDARs-mediated excitotoxicity also contributed to the progressive degeneration of nigral DA neurons in PD (Ambrosi et al., 2014). Research has showed that ligand-gated ion channel NMDARs were vital in the glutamate-induced excitotoxicity in primary dopaminergic cell culture (Oster et al., 2014). Furthermore, glutamate exposure induced Parkin accumulation at mitochondria in a calcium- and NMDARs-dependent manner (Van Laar et al., 2015). It has been confirmed that SNpc DA neurons express functional triheteromeric NMDARs composed of NR1, NR2B and NR2D subunits (Jones and Gibb, 2005). A selective decrease of NR1 pan mRNA levels in layer IV of frontal cortex was found in PD patients (Meoni et al., 1999). However, NR1 expression increased in surviving SN DA neurons from PD brains compared with neurons from controls (Schiemann et al., 2012). Meanwhile, intracellular Ca^2+^ overload triggered by activation of NMDARs is believed to be responsible for inducing of nitric oxide synthases (NOS) activity, affecting mitochondrial integrity and functions (Yamauchi et al., 1998; Sattler and Tymianski, 2000). It has been reported that NMDARs antagonists were considered as potential therapies for patients with PD (Little and Brown, 2014). Among the NMDARs antagonists, memantine has been reported to preferentially blocks extrasynaptic NMDARs of SNpc DA neurons in slices of rat midbrain using whole-cell patch-clamp recordings (Wu and Johnson, 2015). This selective effect on NMDARs might be protective due to the neurotoxic effect of extrasynaptic NMDARs.

## Dysfunction of Iron Metabolism and NMDARs in AD

AD is a progressive neurodegenerative disorder, clinically characterized by a progressive loss of cognitive abilities and dementia, which is closely related to a degree of neuronal and synaptic loss (Selkoe, 1996; Hardy, 1997; Mattson, 1997). The key features of the disease are the accumulation of extracellular amyloid-β (Aβ) plaques and neurofibrillary tangles inside neurons. Although genetic and non-genetic factors are involved in the etiology of AD, the exact reason is still unknown. Increasing evidence suggests that iron might play an important role in the development or progression of AD (Mandel et al., 2007; Ward et al., 2014; Belaidi and Bush, 2016; Van Bergen et al., 2016). The first evidence is that iron accumulates in the same brain regions which are characterized by Aβ deposition such as hippocampus, parietal cortex and motor cortex in AD patients (Dedman et al., 1992; Good et al., 1992). High levels of iron have also been reported in the amyloid plaques in PS/amyloid precursor protein (APP) and APP [V717I] transgenic mice (Falangola et al., 2005), resembling those seen in the brains of AD patients. In addition, higher cortical iron was associated with increased Aβ-plaque-load in mild cognitive impairment (MCI; Van Bergen et al., 2016). Another important link between iron and AD is based on the observation that APP expression is iron-regulated. APP is a single transmembrane metalloprotein that is cleaved to generate the 40–42-amino-acid Aβs by β- and γ-secretases. The existence of a functional IRE in the 5′- UTR of APP mRNA makes it possible to be controlled at the level of mRNA translation by the action of IRE/IRP’s response to iron (Rogers et al., 2002). This implied that high intracellular iron levels could cause increased APP translation and Aβ formation via this mechanism. In addition, iron could induce Aβ precipitation (Mantyh et al., 1993; Huang X. et al., 2004) and significantly enhance the toxicity of Aβ in cultured neuronal cells, whereas iron chelators protect the neurons from Aβ toxicity (Schubert and Chevion, 1995). This provided a direct link between excessive iron and loss of neuronal function seen in AD patients.

However, the underlying mechanisms involved in disturbance of iron homeostasis in AD brain remain unclear. It is reported that Aβ is a metalloprotein that binds transition metal ions through three histidine residues (His6, His13 and His14) located in the N-terminal domain (Nakamura et al., 2007; Altamura and Muckenthaler, 2009). And other protein that accumulates in AD such as tau protein also possesses metal-binding sites (Perry et al., 2003). These might account for the iron accumulation in the affected brain regions in AD. Furthermore, it is reported that DMT1 was colocalized with Aβ in the plaques of postmortem AD brain and the levels of DMT1 was significantly increased in the cortex and hippocampus in APP/PS1 transgenic mouse model compared with wild type-control (Zheng et al., 2009). And the mean serum p97 concentration was elevated 3- to 4-fold in patients with AD as compared to non-AD dementia and normal controls (Kim et al., 2001). In addition, HO-1 is increased in neurofibrillary tangles, senile plaque neurites, granulovacuolar degeneration and neuropil threads in human AD brains (Smith et al., 1994; Perry et al., 2003), suggesting the redistribution of iron due to release from heme proteins in affected areas of the AD brain.

NMDARs–mediated excitotoxicity also contributed to the pathology of AD. Evidence proved that impaired glutamate uptake in astrocytes and neurons in AD can lead to increased concentrations of glutamate at the synapse, which can subsequently trigger NMDARs–mediated excitotoxicity via increase of intracellular Ca^2+^ concentrations (Greenamyre et al., 1988; Tong et al., 2017). It has been shown that extrasynaptic NMDARs were largely associated with NMDARs-mediated excitotoxicity in AD (Hardingham and Bading, 2010). Prolonged activation of extrasynaptic NMDARs increases APP processing, leads to neuronal Aβ release, and ultimately results in AD pathology (Bordji et al., 2010). It has been reported that an open channel blocker memantine could preferentially antagonize excessively activated NMDARs (Lipton, 2004). *In vitro* and *in vivo* studies have demonstrated the neuroprotective effect of memantine in AD models. Memantine could prevent oligomeric Aβ-induced oxidative stress in mature hippocampal neurons (De Felice et al., 2007). More interestingly, memantine decreased the levels of secreted APP and Aβ peptide in human neuroblastoma cells (Ray et al., 2010) and lowered cortical levels of Aβ1–42 in APP/PS1 transgenic mice (Alley et al., 2010). This indicated the important role of excessively activated NMDARs in AD.

## Interaction of Iron and NMDARs Activation

### NMDARs Activation Promoted Iron Accumulation

Results revealed that activation of NMDARs significantly promoted Fe^2+^ entry into cells (Cheah et al., 2006). It was reported that NMDARs activation might promote iron accumulation by accelerating DMT1-mediated iron influx (Cheah et al., 2006), enhancing iron releasing from lysosome and regulation the expression of DMT1 (White et al., 2016). Elevated intracellular iron then aggravated iron-induced cell damage.

### NMDARs Activation Increased NTBI Influx

It has been demonstrated that ferrous iron could block the influx of Ca^2+^ across NMDARs channels in cultured neurons (Nakamichi et al., 2004). This indicated that Fe^2+^ competed with Ca^2+^ for NMDARs to enter primary neurons. This iron entry can be harmful for neurons during aging due to increased NTBI levels. An investigation with interest to the involvement of NMDARs in NTBI influx pathways showed that activation of NMDARs significantly promoted Fe^2+^ entry into cells using fluorescence-based single cell analysis in rat hippocampal primary cultures (Pelizzoni et al., 2011). This elevation of iron was accompanied by a corresponding increase in reactive oxygen species (ROS) production and higher susceptibility of neurons to death.

Another investigation demonstrated a novel signaling cascade for glutamate in regulating iron uptake in the brain (Cheah et al., 2006). It is increasingly appreciated that glutamate via NMDARs triggers calcium influx, then activates neuronal NOS (nNOS) to produce NO, which causes excitability toxicity (Guix et al., 2005; Cheah et al., 2006; Chen et al., 2013; Courtney et al., 2014). Research has identified a signaling cascade whereby NMDARs regulated iron homeostasis (Cheah et al., 2006). Their results showed that the activation of NMDARs could increase intracellular iron levels in PC12 cells via NO-Dexras1-peripheral benzodiazepine receptor-associated protein 7 (PAP7)-DMT1 signaling cascade to enhance iron uptake (Cheah et al., 2006). Additionally, chelation of intracellular iron blocked formation of free radicals in brain cultures and also markedly attenuated NMDA neurotoxicity (Cheah et al., 2006). Furthermore, subsequent investigation showed that Dexras1 was required for NMDA-elicited neuronal toxicity via NO and iron influx (Chen et al., 2013). This implies that NMDA-NO activation-induced iron uptake might play an important role in neurotoxicity and misregulation of this pathway might participate in iron accumulation in PD. It has been shown that nNOS binding to CAPON leading to NO delivery to Dexras1 and S-nitrosylation of Dexras1. Then S-nitrosylation of Dexras1 could enhance iron uptake by regulating the function of iron importer DMT1. It was also reported that Dexras1 could be phosphorylated by protein kinase A (PKA) on serine 253. This PKA activation reduced Dexras1 S-nitrosylation, leading to an inhibition of iron influx (Chen et al., 2015). This indicated the important role of Dexras1 S-nitrosylation in iron uptake. In addition, PAP7 might presumably serve as a scaffold delivering Dexras1 into proximity to DMT1 (Cheah et al., 2006). However, another research showed that PAP7 played a role in cellular iron metabolism. They found that PAP7 was internalized in parallel with the internalization of DMT1 following iron feeding. And downregulated PAP7 expression in K562 cells with small interfering RNA led to downregulation of DMT1 (IRE) protein but not DMT1 (−IRE) mRNA. However, they did not measure iron uptake after downregulation of PAP7 expression. The levels of TfR1 and ferritin were not affected following transfection with siPAP7, indicating that the intracellular iron level might not change (Okazaki et al., 2012). Moreover, they also reported that overexpression of PAP7 had no effect on DMT1 (IRE) expression which is consist with the results of Cheah et al. (2006). Further research should be conducted to figure out the mechanisms underlying the effect of PAP7 on DMT1 and DMT1-induced iron uptake.

Rhes (Dexras2), a homolog of Dexras1, is a highly conserved small GTP binding protein belonging to the Ras superfamily. Rhes shares 67% identity with Dexras1. Although the physiological role of Rhes is not fully understood, results showed that Rhes physiologically interacted with PAP7 and participated in DMT1-induced iron uptake via pathway similar to Dexras1 (Choi et al., 2013). However, the mechanisms underlying the effect of Rhes on DMT1-induced iron uptake are different from Dexras1. They showed that Rhes was not S-nitrosylated after NO-treatment, however was phosphorylated by PKA at serine-239 (Choi et al., 2013). Rhes is selectively localized to the corpus striatum (Errico et al., 2008), so might involved in NMDARs-induced iron entry into the striatal neurons and maintain striatal iron homeostasis via PKA-Rhes-DMT1 pathway. In addition, striatum received nigral dopaminergic synapses projection and is responsible for movement balance. Imbalance of iron metabolism in striatum might contribute to the degeneration of striatal neurons and dysregulation of dopaminergic function in the striatum due to iron-induced oxidative stress, then affecting motor function.

### NMDARs Activation Enhanced Iron Releasing from Lysosome

In fact, Dexras1 also could regulate TfR-mediated iron uptake as well as NTBI uptake (DMT1-mediated iron uptake) (Cheah et al., 2006). However, the exact mechanisms underlying this effect are not elucidated. Subcellular localization experiments showed that DMT1 + IRE had higher surface expression and was internalized from the plasma membrane with slower kinetics than DMT1 − IRE. And it was not efficiently recycled and was targeted to lysosomes. While DMT1 − IRE is efficiently sorted to recycling endosomes upon internalization (Lam-Yuk-Tseung and Gros, 2006). This implicated that different isoforms of DMT1 might have different functions on regulation of the subcellular localization of Fe^2+^ transport. As lysosomal iron serves as a main source for intracellular iron and DMT1 plays a role in iron recycling from lysosome to cytoplasm, DMT1-mediated iron release from lysosome might be responsible for increased intracellular iron levels. Findings from patch-clamping of individual lysosomes showed that they could transmit iron through their membrane (Dong et al., 2008). Recently, an investigation confirmed that the Dexras1/ACBD3(PAP7)/DMT1 complex was located on the lysosomes (White et al., 2016). Collapsing the proton gradient or blocking DMT1 channel both could reduce cytosolic iron pool (White et al., 2016), indicating that Dexras1/ACBD3/DMT1 complex played roles in iron release from lysosome. Therefore, NMDA activation not only led to Dexras1/DMT1 mediated increase in iron uptake, but also enhanced Dexras1-dependent iron release from lysosome.

### NMDARs Activation Increased DMT1 Expression

Activation of NMDARs not only could affect the iron uptake function of DMT1, but also could regulate its expression. It has been reported that mRNA expression of DMT1-1B and DMT1 + IRE increased after 5 min exposure of 50 μM NMDA to primary hippocampal cultures, but not mRNA expression of DMT1 − IRE (Haeger et al., 2010). NMDA also enhanced DMT1 protein expression, which was abolished by the transcription inhibitor actinomycin D and NMDARs antagonist MK-801 (Haeger et al., 2010). This stimulation on iron entry pathway via DMT1 could ensure an adequate iron supply. This is of potential importance because iron deficiency hinders learning processes and impairs cognitive performance (Carlson et al., 2009; Muñoz et al., 2011; Estrada et al., 2014). This implicated that NMDARs-activation stimulated expression of the iron transporter DMT1-1B + IRE, which presumably played a significant role in hippocampal spatial memory formation. Interestingly, both excessive activation of NMDARs and upregulation of DMT1 were contributing factors in degeneration of DA neurons in PD, indicating the link between activation of NMDARs and upregulation of DMT1 in PD. Further studied should be conducted to elucidate this possibility and the underlying mechanisms.

### NMDARs-Induced Co-Activation of ATP-Sensitive Potassium (K_ATP_) Channels Might Promote Iron Influx via DMT1

K_ATP_ channel was first discovered by Akinori Noma in cardiac myocytes and was demonstrated to be important for regulation of cellular energy metabolism in the control of membrane excitability (Noma, 1983). Liss et al. (2005) provided evidence that selective activation of K_ATP_ channels of DA midbrain neurons in the SN was a potential mechanism for the selective degeneration of DA neurons in PD. Increased activity of K_ATP_ channels due to metabolic stress leads to membrane potential hyperpolarization and reduced SN DA activity (Liss et al., 2005). Although in adult mice K_ATP_ channels in DA neurons of both SN and VTA are formed by the same subunits (four Kir6.2 subunits and four regulatory SUR1 subunits), the activation of nigral K_ATP_ channels of DA neurons was responsible for the selective degeneration of DA neurons in PD (Liss et al., 2005). Moreover, genetic inactivation of the pore-forming subunit Kir6.2 of K_ATP_ channels induced a selective rescue of SN DA (but not VTA DA) neurons in MPTP-induced PD models (Liss et al., 2005). This indicated that activation of nigral K_ATP_ channels contributed to the selective degeneration of DA neurons in the SN.

It is reported that the activation of K_ATP_ channels induced hyperpolarization of the membrane potential of DA neurons in the SN following MPP^+^ treatment (Liss et al., 2005). Furthermore, it was reported DMT1-mediated iron transport was driven at higher rates of hyperpolarized potentials (Gunshin et al., 1997). This makes the possibility that the hyperpolarization of cell membrane induced by activation of K_ATP_ channels might increase DMT1-mediated iron transport, and enhance iron influx into the DA neurons. Results in our experiments demonstrated that activation of the K_ATP_ channels in the DA cells caused hyperpolarization of the cell membrane and subsequently enhanced ferrous iron uptake function (Du X. et al., 2016). Results showed that activation of K_ATP_ channels resulted in increased free iron levels in the SK-N-SH cells and this was partially blocked by DMT1 knockdown. Further studies showed that activation of K_ATP_ channels by diazoxide prolonged Fe^2+^-evoked currents in DMT1-transfected HEK293 cells using whole cell patch clamp recordings (Du X. et al., 2016). And inhibition of the K_ATP_ channels protected the DA neurons from the ferrous iron insult (Du X. et al., 2016). These results suggest that the activation of K_ATP_ channels could enhance DMT1-mediated iron uptake, resulting in an increased intracellular iron contents and oxidative stress, and ultimately cell damage.

It has been proved that there is a relationship between NMDARs and K_ATP_ channels. Elevated mRNA expressions of K_ATP_ channels and NMDARs subunits were found in human SN DA neurons from PD patients (Schiemann et al., 2012). NMDARs stimulation *in vitro* induced the co-activation of K_ATP_ channels in subthalamic neurons (Shen and Johnson, 2010). Activity of K_ATP_ channels in medial DA neurons of the SN enabled NMDA-mediated bursting *in vitro* and *in vivo* in anesthetized mice (Schiemann et al., 2012). Furthermore, activation of K_ATP_ channels in already metabolically challenged SN DA neurons could promote excitotoxicity and increase NMDARs-mediated calcium loading (Schiemann et al., 2012). Calcium-triggered ROS production from mitochondria in turn activated K_ATP_ channels in highly vulnerable SN DA neurons (Liss et al., 2005). In addition, NMDARs-mediated NO production might activate K_ATP_ channels in large DRG neurons via direct S-nitrosylation of SUR1 subunit (Kawano et al., 2009). The link of K_ATP_ channels and DMT1 indicated that co-activation of NMDARs and K_ATP_ channels could affect iron uptake function of DMT1, which might contribute to iron accumulation and the degeneration of neurons in neurological diseases. Further investigation should be conducted in the future to reveal the underlying mechanisms.

### Iron Affected NMDARs-Mediated Synaptic Transmission

It was reported that iron has an effect on NMDARs-mediated synaptic plasticity. Investigation demonstrated that glutamatergic neurotransmission pathways were regulated by dietary iron (Han and Kim, 2015). They showed that NMDARs were significantly elevated in the prefrontal cortex and hippocampus of iron-loaded rats. These findings indicate the important role of iron in learning and memory via regulation NMDARs-mediated neurotransmission. In addition, study showed that intracellular iron signaling could modulate neuronal excitability in the hippocampus (White et al., 2016). They showed that iron was released in neurons from lysosome can regulate NMDARs mediated glutamatergic excitability via PKC/Src/NR2A pathway (White et al., 2016). This indicates that iron can act in a feedback manner to modulate NMDA function and thus maintain the excitability of neurons in normal condition. In addition, NMDARs activation induce rapid opening of Ca^2+^ channel across cell membranes, followed by an increase in free Ca^2+^ ions in the cytoplasm and subsequent signaling cascade in the CNS (Paul and Connor, 2010). Hippocampal neurons require iron to generate RyR-mediated calcium signals after NMDARs stimulation, which in turn promotes ERK1/2 activation, an essential step of sustained LTP (Muñoz et al., 2011). It was reported that iron stimulated RyR-mediated Ca^2+^ release from endoplasmic reticulum (ER) in PC12 cells (Múñoz et al., 2006). Further study showed that iron-induced ROS generation is required for RyR-mediated Ca^2+^ release for long-term potentiation in primary hippocampal neurons (Muñoz et al., 2011).

However, evidence has also proved that iron was involved in glutamate excitotoxicity (Yu et al., 2009). As is known that glutamate excitotoxicity could be induced by threohydroxyaspartate (THA), which could inhibit glutamate uptake and lead to accumulation of synaptic glutamate and over stimulation of the postsynaptic receptors. It was found that increased iron level was involved in THA-induced glutamate excitotoxicity in rat spinal cord tissue (Yu et al., 2009). And iron chelator deferoxamine (DFO) could completely prevent THA-induced motor neuron degeneration. Importantly, NMDARs inhibitor MK-801 could inhibit glutamate and hypoxia/reperfusion-mediated damage, but not the kainate/AMPA antagonist CNQX (Yu et al., 2009). This supports the idea that iron contributed to glutamate-induced excitotoxicity via NMDARs and regulation of iron level might be an effective strategy for protection against glutamate-induced excitotoxicity. Therefore, iron is required for NMDARs-mediated synaptic plasticity in physiologic conditions. However, iron overload might promote NMDARs-mediated excitotoxicity possibly by enhancing RyR-mediated Ca^2+^ release and calcium-induced cell damage. Further studies should be conducted to investigate this possibility and the underlying mechanisms.

## Conclusion and Perspectives

**Figure 1:**
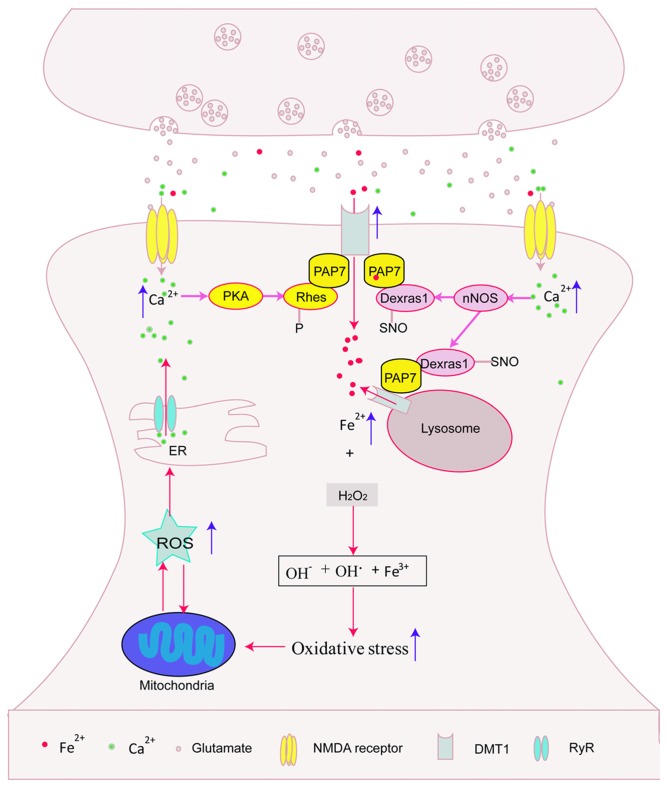
**Possible interaction between N-methyl-D-aspartate receptors (NMDARs) activation and iron overload.** NMDARs activation might promote divalent metal transporter 1 (DMT1)-mediated iron influx via protein kinase A (PKA)-Rhes-DMT1, NO-Dexras1-peripheral benzodiazepine receptor-associated protein 7 (PAP7)-DMT1 signaling cascade pathway and enhance iron releasing from lysosome, then aggravated iron-induced cell damage. In addition, iron overload might stimulate calcium release from endoplasmic reticulum (ER). This indicated that glutamate-induced neurotoxicity and iron participated to a vicious cycle of neuronal death.

In this review article, we describe the possible relationship between NMDARs activation and iron deposit, which both contributed to the pathogenesis of neurological diseases including PD and AD. NMDARs activation might promote iron accumulation by enhancing DMT1-mediated iron influx, stimulating iron releasing from lysosome, and regulating DMT1 expression, thus aggravating iron-induced cell damage. On the other hand, iron overload might aggravate NMDARs-mediated glutamate excitotoxicity. This suggests that glutamate-induced neurotoxicity and iron participate to a vicious cycle of neuronal death (Figure 1). Therefore, regulation of iron levels might be effective for protection against glutamate-induced excitotoxicity and NMDARs might be a potential target for the treatment of iron-induced neurodegenerative processes. The drug discovery strategy is being oriented toward the development of new molecules targeting both iron overload and activation of NMDARs. However, NMDARs-mediated excitotoxicity and iron deposit were also reported in other neurological diseases such as stroke, Huntington’ disease (Carvajal et al., 2016; Li and Reichmann, 2016; Petrova et al., 2016). The exact mechanisms underlying interaction between NMDARs activation and iron accumulation in these diseases should be conducted in the future. The future of this research might have significant impact on the clinical treatment of these neurological diseases.

## Author Contributions

HX wrote the manuscript. HJ and JX revised the manuscript.

## Conflict of Interest Statement

The authors declare that the research was conducted in the absence of any commercial or financial relationships that could be construed as a potential conflict of interest. The reviewer DT and handling Editor declared their shared affiliation, and the handling Editor states that the process nevertheless met the standards of a fair and objective review.
